# Metabolic modeling of synthesis gas fermentation in bubble column reactors

**DOI:** 10.1186/s13068-015-0272-5

**Published:** 2015-06-20

**Authors:** Jin Chen, Jose A. Gomez, Kai Höffner, Paul I. Barton, Michael A. Henson

**Affiliations:** Department of Chemical Engineering, University of Massachusetts, Amherst, MA 010003 USA; Process Systems Engineering Laboratory, Department of Chemical Engineering, Massachusetts Institute of Technology, Cambridge, MA 02139 USA

**Keywords:** Metabolic modeling, Bioprocess engineering, Microbial fermentation, Ethanol production

## Abstract

**Background:**

A promising route to renewable liquid fuels and chemicals is the fermentation of synthesis gas (syngas) streams to synthesize desired products such as ethanol and 2,3-butanediol. While commercial development of syngas fermentation technology is underway, an unmet need is the development of integrated metabolic and transport models for industrially relevant syngas bubble column reactors.

**Results:**

We developed and evaluated a spatiotemporal metabolic model for bubble column reactors with the syngas fermenting bacterium *Clostridium ljungdahlii* as the microbial catalyst. Our modeling approach involved combining a genome-scale reconstruction of *C. ljungdahlii* metabolism with multiphase transport equations that govern convective and dispersive processes within the spatially varying column. The reactor model was spatially discretized to yield a large set of ordinary differential equations (ODEs) in time with embedded linear programs (LPs) and solved using the MATLAB based code DFBAlab. Simulations were performed to analyze the effects of important process and cellular parameters on key measures of reactor performance including ethanol titer, ethanol-to-acetate ratio, and CO and H_2_ conversions.

**Conclusions:**

Our computational study demonstrated that mathematical modeling provides a complementary tool to experimentation for understanding, predicting, and optimizing syngas fermentation reactors. These model predictions could guide future cellular and process engineering efforts aimed at alleviating bottlenecks to biochemical production in syngas bubble column reactors.

## Background

The development of alternative, renewable sources of fuels and chemicals to reduce our dependence on petroleum has emerged as a paramount challenge for maintaining the economic security and environmental wellbeing of the USA. An essential component of this quest is to develop renewable, environmentally friendly sources of biochemicals via the conversion of readily available plant biomass and waste streams that represent a significant quantity of reduced carbon feedstock. An emerging conversion route with wide feedstock versatility is direct fermentation of waste gas streams and synthesis gas (syngas; mainly comprised of H_2_/CO/CO_2_) by specialized CO fermenting microbes. Because syngas can be produced relatively cheaply from a wide variety of biomass feedstocks [1, 2], the bottleneck in this route is the syngas fermentation step. Key considerations are the metabolic capabilities and performance of the microbial catalyst that converts syngas into the desired biochemical, gas–liquid mass transfer characteristics that determine the availability of soluble gas components for microbial conversion and the bioreactor design that affects all aspects of the conversion process.

### Gas fermentation for production of fuels and chemicals

A model syngas-consuming organism is *Clostridium ljungdahlii*, a rod-shape anaerobic bacterium that was discovered in 1987 and found to have the ability to ferment CO and H_2_ into ethanol and acetate [3]. This discovery initiated a wave of research and development efforts aimed at understanding and optimizing syngas fermentation for ethanol production [4]. Several other bacteria including *C. aceticum* [5], *Acetobacterium woodii* [6], and *C. carboxidivorans* [7] also have been studied for syngas fermentation. All these mesophilic bacteria synthesize ethanol through the reductive acetyl-CoA metabolic pathway, a non-cyclic, fermentative pathway which is active under anaerobic conditions [8]. Electrons required in the pathway are supplied by the syngas components CO and H_2_. Optimal growth conditions for *C. ljungdahlii* have been reported as 37 °C and pH of 6.0 [9], but at least one study claims that ethanol synthesis was increased at lower pH values where growth was significantly reduced [10].

One of the most challenging problems in syngas fermentation is to establish culture conditions which offer favorable gas–liquid mass transfer characteristics such that the syngas is readily dissolved and available for microbial conversion. A variety of reactor types including stirred tank reactors, trickle bed reactors, packed bed reactors, monolithic biofilm reactors, membrane-based reactors, and bubble column reactors have been investigated [9]. While more advanced designs based on bubble column reactors are being developed for large-scale production [8], most academic research has been performed in stirred tank reactors with continuous liquid and syngas flows. Stirred tank reactors can have CO mass transfer coefficients over 100 h^−1^ through the use of specially designed impellers, high agitation rates, and microspargers that create small gas bubbles [9, 11]. However, substantially enhanced syngas mass transfer can be achieved in bubble column reactors due to higher average mass transfer driving forces caused by favorable gas composition spatial profiles and longer gas–liquid contact times. Another potential strategy for increasing syngas solubility is the use of elevated operating pressures [12]. This approach has not been widely studied because gas compression at the industrial scale is costly.

**Fig. 1:**
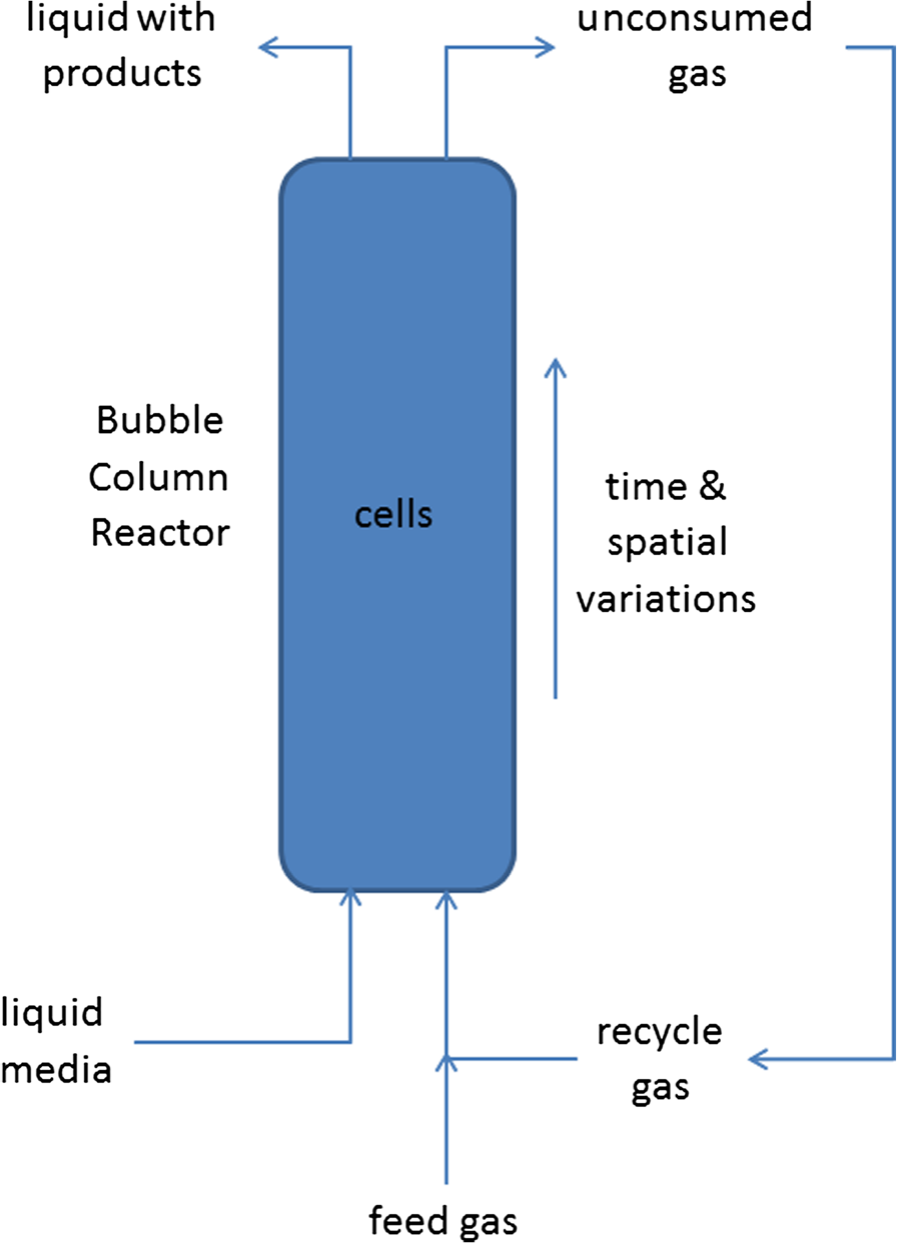
Bubble column reactor for microbial syngas fermentation

**Fig. 2:**
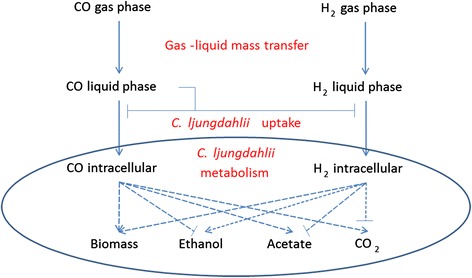
The effects of CO and H_2_ mass transfer and cellular uptake on biomass production and the distribution of metabolic products by *C. ljungdahlii*. The lines with arrows represent positive/activating effects and the lines with bars represent negative/inhibitory effects

Because the syngas feed is introduced into the bottom of the bubble column (Fig. 1), CO and H_2_ concentrations decrease as the gas flows up the column due to cellular consumption. Therefore, the column has spatially varying dissolved gas concentrations that affect cellular growth and product synthesis. In principle, high dissolved CO concentrations throughout the column are desirable since CO is the primary carbon source for growth. Previous experimental studies [8, 13] have suggested that high dissolved CO levels can inhibit both CO and H_2_ uptake rates (Fig. 2). Therefore, column optimization requires that dissolved CO concentrations are sufficiently high near the top of the column to promote growth, but CO concentrations near the bottom of the column are not so high as to significantly inhibit gas uptake rates. The relative amounts of dissolved CO and H_2_ have a strong impact on the split between the desired product ethanol and the undesired byproduct acetate [14, 15]. While ethanol synthesis is promoted at high H_2_ concentrations, the ratio of ethanol to acetate increases with increasing H_2_ concentration. Therefore, the objective is to establish desirable H_2_ and CO concentration profiles along the column such that the ethanol production is maximized and the acetate production is minimized. The design and operation of bubble column reactors to achieve a suitable compromise between these competing objectives has proven to be a difficult challenge that has limited commercial syngas fermentation technology. We believe that the development of model-based techniques for simulating and optimizing syngas bubble column reactors is essential to advance this technology.

### Spatiotemporal modeling of microbial metabolism

Steady-state [16] and dynamic [17–20] flux balance analysis techniques based on genome-scale metabolic reconstructions have become standard tools for analyzing microbial metabolism. Recently, the first genome-scale metabolic reconstruction of a CO fermenting organism was introduced for the bacterium *C. ljungdahlii* [21]. The iHN637 model includes an extensive reaction network of central metabolism, including the pathways involved in carbon fixation and energy conservation. The model was shown to be capable of producing acetate, ethanol, and 2,3-butanediol under conditions consistent with experimental data. While a few very simple unstructured growth models of syngas fermentation have been developed [22, 13], we are not aware of any dynamic models based on FBA and/or genome-scale metabolic reconstructions.

Dynamic modeling of syngas bubble column reactors poses an additional challenge not encountered in well-mixed stirred tank reactors. Namely, spatial gradients are present because the dissolved CO and H_2_ concentrations decrease as the gas flows up the column due to cellular consumption [8]. The cellular growth and product synthesis rates along the column are determined by these local dissolved gas concentrations. While spatiotemporal models that account for both spatial and temporal variations in the extracellular environment have been constructed, these studies utilized table lookups of precomputed FBA solutions [23–25] or heuristic lattice-based descriptions of nutrient diffusion [23–25]. We propose a general methodology for spatiotemporal metabolic modeling based on combining genome-scale reconstructions with fundamental transport equations that capture the relevant convection and/or diffusional processes. Additional details on the numerical solution procedure will be available in a forthcoming publication. The model solution procedure involved spatially discretizing the partial differential equations (PDEs) to generate an ordinary differential equation (ODE) system with embedded linear programs (LPs) that was integrated with DFBAlab [26], a MATLAB code that performs reliable and efficient dynamic FBA simulations. We demonstrated the capabilities of our approach by performing dynamic simulations with the syngas bubble column reactor model presented below. The contributions of the present study include the following: (1) a detailed presentation of the bubble column model including experimentally derived parameters and (2) an extensive investigation into the effects of process and cellular parameters on bubble column performance.

## Results and discussion

### Impact of design and operating parameters on bubble column performance

The model was used to predict the effects of important design and operating parameters on bubble column performance, as measured by the liquid and gas phase concentrations exiting the reactor under steady-state conditions. Due to the lack of accurate dissolved gas uptake kinetic parameters and directly comparable experimental data for model validation, the model predictions should be viewed as qualitative rather than quantitative. This capability was deemed sufficient for predicting trends with respect to key parameters. Each prediction was generated by simulating bubble column startup with *N* = 100 node points and a final time of 1000 h to obtain the steady-state solution. Typically 5–10 simulations were performed for each parameter, and plots showing parameter trends were generated by linearly interpolating the cases ran (indicated by asterisks) within MATLAB.

**Fig. 3:**
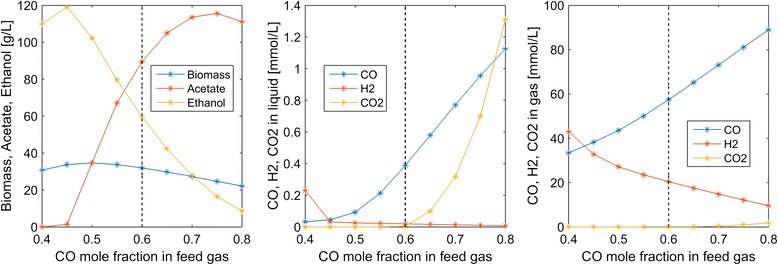
Effect of the feed CO mole fraction on steady-state concentrations in the exiting liquid and gas streams. The *dashed lines* indicate the nominal feed CO mole fraction used in the other simulations

We first investigated the impact of the feed composition by varying the CO mole fraction with the H_2_ mole fraction adjusted such that the mole fractions summed to unity. Experimental studies [27] have shown that ethanol synthesis is favored relative to acetate synthesis at high H_2_/CO feed ratios. We observed the same trend in our bubble column simulations (Fig. 3). The ethanol titer was predicted to achieve a maximum of 120 g/L at a CO mole fraction of 0.45, which represents a H_2_ rich feed. As the mole fraction was increased beyond this value, the ethanol concentration was predicted to decrease and acetate synthesis began. The ethanol and acetate concentrations were approximately equal at a mole fraction of 0.55. Thereafter, the acetate titer increased rapidly, the ethanol titer decreased rapidly, and CO_2_ synthesis began due to low dissolved H_2_ levels. Interestingly, the acetate concentration decreased at CO mole fractions beyond 0.75, presumably due to reduced biomass production. A maximum biomass concentration of about 35 g/L was predicted for a 50/50 CO/H_2_ mixture.

**Fig. 4:**
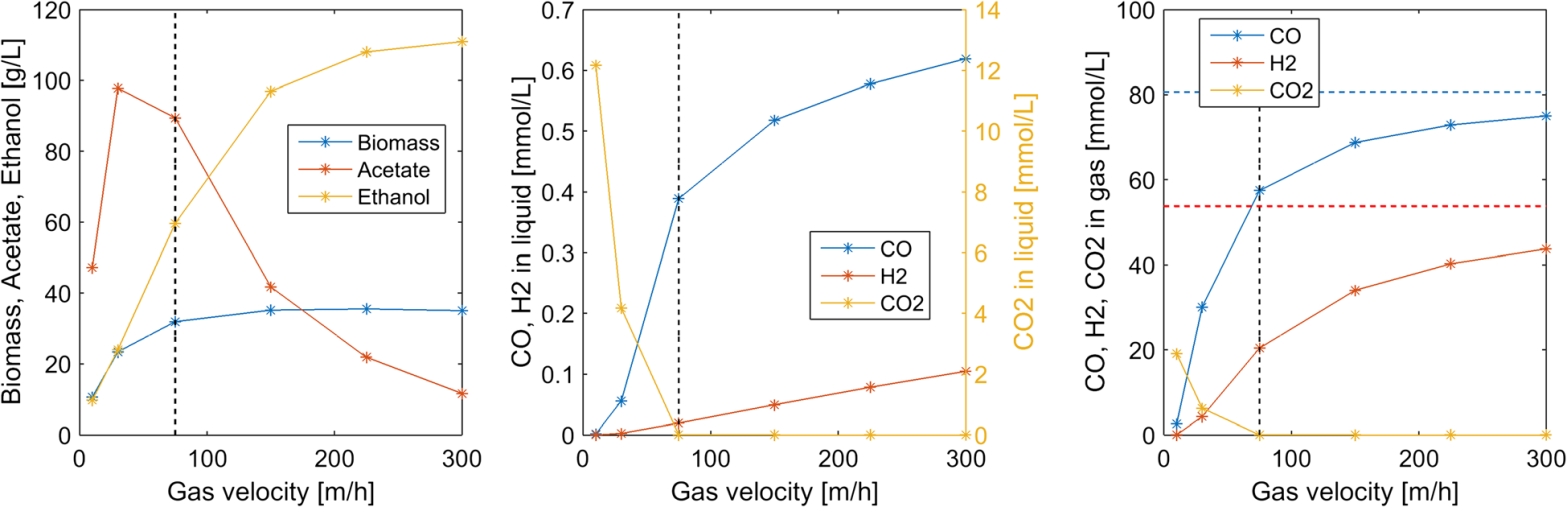
Effect of the superficial gas velocity on steady-state concentrations in the exiting liquid and gas streams. The *vertical dashed lines* indicate the nominal superficial gas velocity used in the other simulations. The *horizontal dashed lines* indicate the inlet gas concentrations of CO and H_2_

Next we explored the impacts of the superficial gas velocity (*u*_*G*_) reactor performance. Increasing *u*_*G*_ also caused the gas volume fraction ε_*G*_ to increase according to Eq. 9. High *u*_*G*_ values were predicted to increase dissolved CO and H_2_ concentrations at the expense of reduced CO and H_2_ conversions in the gas phase (Fig. 4). Due to enhanced dissolved H_2_ concentrations, high *u*_*G*_ values produced more favorable ethanol/acetate splits. For example, *u*_*G*_ = 300 m/h produced an ethanol/acetate ratio of 10:1 but CO and H_2_ conversions of only 7 and 19 %, respectively. Conversely, low *u*_*G*_ values produced more favorable conversions but high acetate concentrations as well as substantial CO_2_ synthesis.

**Fig. 5:**
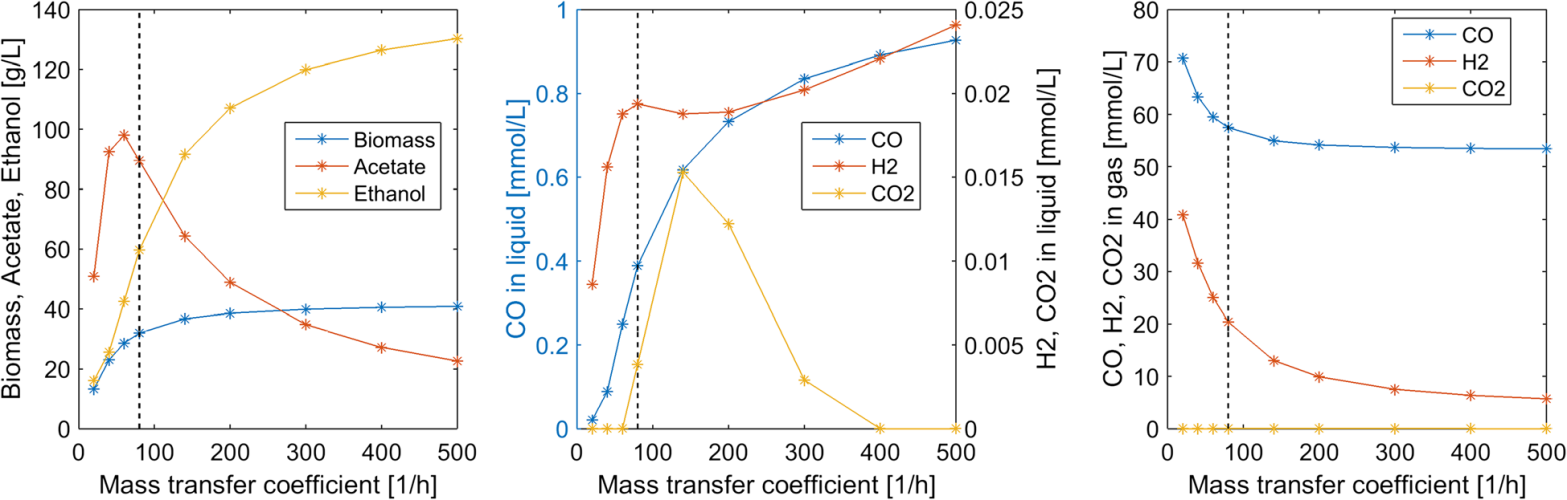
Effect of the CO gas–liquid mass transfer coefficient *k*_*m*,*C*_ on steady-state concentrations in the exiting liquid and gas streams. The H_2_ and CO_2_ mass transfer coefficients were set to be 2.5*k*_*m*,*C*_ and *k*_*m*,*C*_, respectively. The *dashed lines* indicate the nominal *k*_*m*,*C*_ value used in the other simulations

Many experimental studies have argued that the efficiency of syngas fermentation is limited by gas–liquid mass transfer [28]. To explore this claim, we varied the CO gas–liquid mass transfer coefficients *k*_*m*,*C*_ to cover a range of values reported in the literature [9]. As with our nominal values, we set the H_2_ mass transfer coefficient *k*_*m*, *H*_ to be 250 % larger than the CO value and the CO_2_ mass transfer coefficient *k*_*m*,*D*_ to be equal to the CO value. As expected, the primary value of high mass transfer coefficients was predicted to increase dissolved CO and H_2_ concentrations, with CO much more strongly affected (Fig. 5). Below our nominal value *k*_*m*,*C*_ = 80 h^−1^, acetate was the primary byproduct and no CO_2_ was synthesized. Above this nominal value, the acetate titer decreased rapidly and the ethanol titer increased rapidly such that the ethanol/acetate ratio was 5.75 at *k*_*m*,*C*_ = 500 h^−1^. Such high mass transfer coefficients can be achieved in bubble column reactors through the use of syngas microsparging and/or internal packing to increase gas–liquid contact [9]. Enhanced gas–liquid mass transfer also improved syngas consumption, with the CO and H_2_ conversions increased to 34 and 89 %, respectively, at *k*_*m*,*C*_ = 500 h^−1^.

**Fig. 6:**
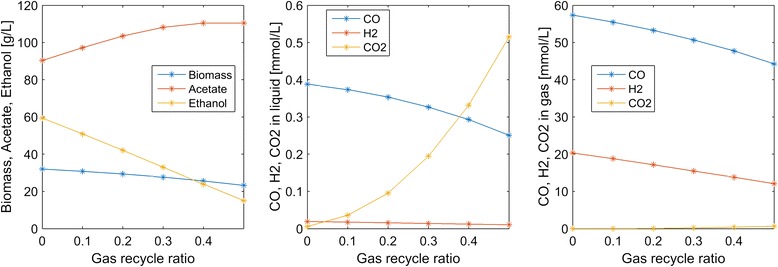
Effect of the gas recycle ratio on steady-state concentrations in the exiting liquid and gas streams. No gas recycle was used in the other simulations

Most column operating conditions investigated in this study were predicted to produce low syngas conversions due to limited gas–liquid mass transfer and cellular uptake rates. For example, our nominal conditions resulted in 62 % H_2_ conversion and 29 % CO conversion. One method for increasing these conversions is partial recycle of unconsumed gas exiting the top of the column (Fig. 1). We explored the effects of gas recycle by allowing a fraction α of the gas exiting the column to be recycled and mixed with the fresh syngas feed. While syngas conversion was predicted to be improved as expected, gas recycling had the undesirable effect of substantially reducing the ethanol titer and the ethanol/acetate ratio (Fig. 6). This behavior seemed to be caused by decreasing dissolved H_2_ concentrations as the recycle ratio was increased.

### Impact of gas uptake parameters on bubble column performance

**Fig. 7:**
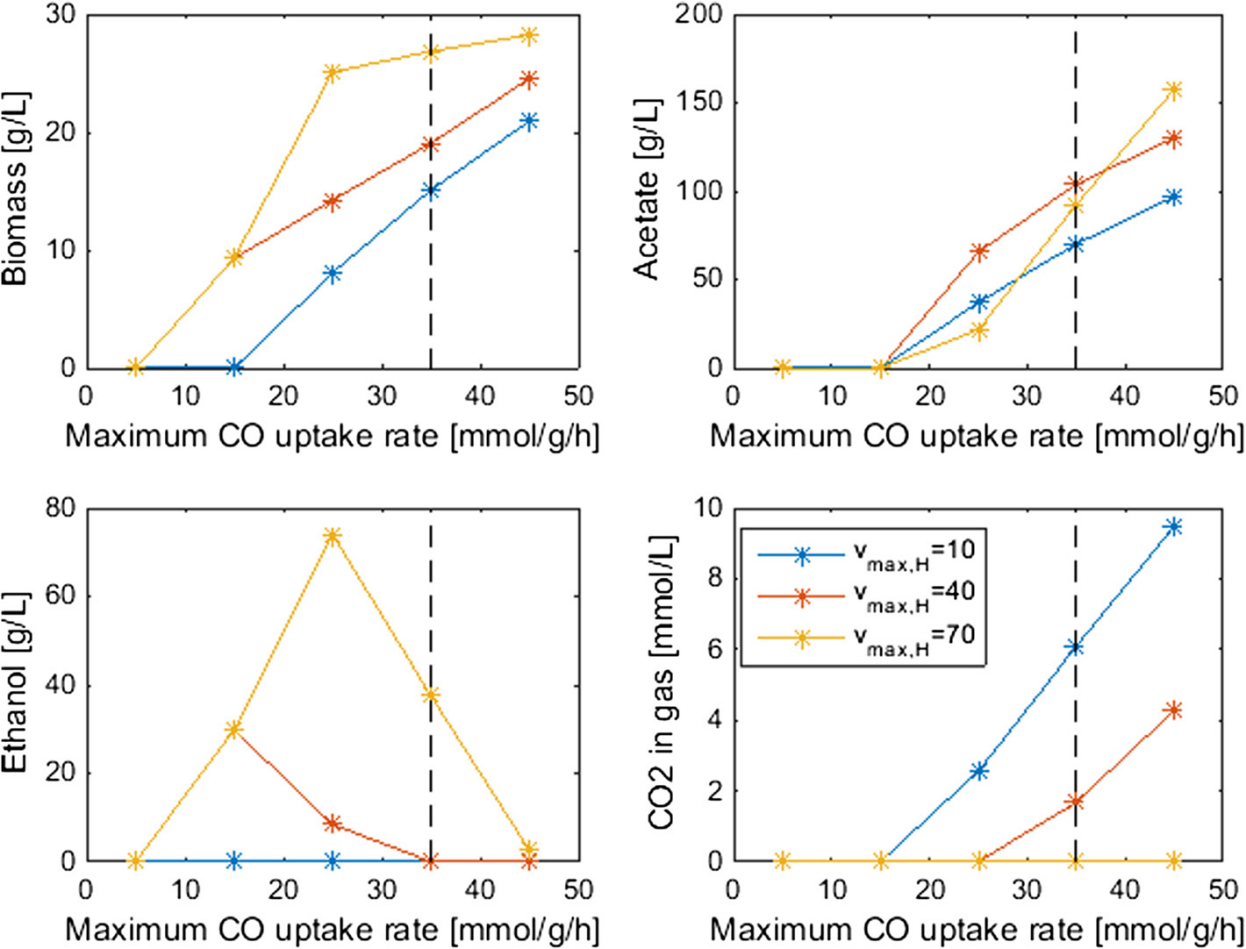
Effect of the CO and H_2_ maximum uptake rate parameters on steady-state biomass and byproduct concentrations at the top column. The *dashed lines* indicate the nominal CO maximum uptake rate used in the other simulations. The nominal H_2_ maximum uptake rate was *v*_*max*_, _*H*_ = 70 mmol/gDW/h

The model was used to predict the effects of important gas uptake parameters on bubble column performance, as measured by steady-state concentrations at the column exit as before. Because our nominal CO and H_2_ maximum uptake rate values had substantial uncertainty, we varied these maximum rates to investigate their impact. Both uptake rate parameters were predicted to have substantial effects on biomass production, with small rates insufficient to meet the ATP maintenance requirements of the cell and generating no growth (Fig. 7). The maximum rate parameters also affected both the amount and distribution of metabolic byproducts. For a H_2_ maximum uptake rate *v*_*max*_, _*H*_ = 10 mmol/gDW/h, the model predicted that no ethanol would be synthesized regardless of the CO uptake rate. In this case, increasing amounts of acetate and CO_2_ were produced as the CO maximum uptake rate *v*_*max*_, _*C*_ was increased. For larger H_2_ maximum uptake rates, increasing amounts of ethanol were synthesized up to *v*_*max*_, _*C*_ = 25 mmol/gDW/h, at which point the ethanol titer began to drop while the acetate titer continued to increase. We also varied the saturation constants in the CO and H_2_ uptake rate expressions (Eq. 2) to examine their impacts. The main effect of increasing the CO saturation constant was to decrease the acetate titer and increase the ethanol titer by establishing more favorable ratios of the two gas uptake rates (results not shown). Decreasing the H_2_ saturation constant had the same effect.

**Fig. 8:**
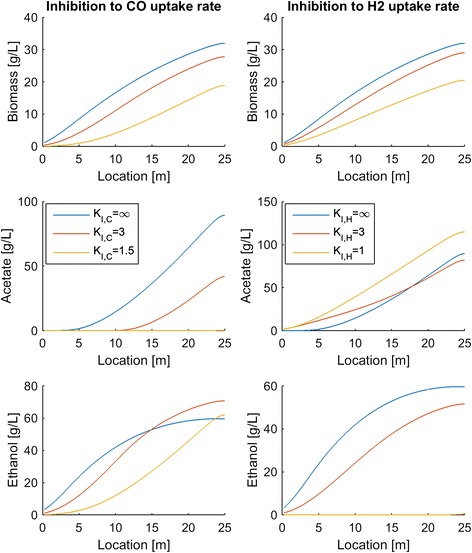
Effect of CO inhibition of CO uptake (*left*) and H_2_ uptake (*right*) on steady-state biomass and byproduct production throughout the column. The nominal case corresponds to no inhibition (*K*_*I*_, _*C*_ = *K*_*I*_, _*H*_ = 10^6^ g/L)

Previous experimental studies [8, 13] have suggested that high dissolved CO levels can inhibit the uptake of CO and/or H_2_. To explore the impact of such inhibitory effects, we modified the uptake rate expressions as follows:

1 $$ \begin{array}{cc}\hfill {v}_C=\frac{v_{max,C}C}{K_{m,C}+C+\raisebox{1ex}{${C}^2$}\!\left/ \!\raisebox{-1ex}{${K}_{I,C}$}\right.}\frac{1}{1+\frac{E+A}{K_I}}\hfill & \hfill {v}_H=\frac{v_{max,H}C}{K_{m,H}+H}\frac{1}{1+\frac{E+A}{K_I}}\frac{1}{1+\frac{C}{K_{I,H}}}\hfill \end{array} $$

where *K*_*I*_, _*C*_ and *K*_*I*_, _*H*_ are parameters that account for CO inhibition of CO uptake and H_2_ uptake, respectively. Each parameter was varied independently to obtain three values that corresponded to no inhibition (*K*_*I*_, _*C*_ = *K*_*I*_, _*H*_ = 10^6^ g/L), moderate inhibition, and strong inhibition. As expected, inhibition of either CO or H_2_ uptake was predicted to reduce steady-state biomass production throughout the column (Fig. 8). CO inhibition had the interesting effect of substantially reducing acetate synthesis but having very little impact on the exiting ethanol titer due to the establishment of more favorable intracellular CO/H_2_ levels. At the highest level of inhibition, no acetate was produced and the ethanol titer was over 60 g/L. Conversely, CO inhibition of H_2_ uptake shifted the product distribution strongly towards acetate with no ethanol produced at the highest inhibition level.

## Conclusions

Bubble columns are the preferred reactor technology for industrial production of fuels and chemicals from synthesis gas. A number of experimental studies have been performed to investigate the effects of the microbial catalyst, the column design parameters, and the column operating conditions on syngas fermentation performance [9]. Because the effect of cellular and process parameters on column performance are complex, mathematical modeling provides a complementary tool to experimentation for understanding, predicting, and optimizing syngas fermentation reactors. We developed a spatiotemporal metabolic model for bubble column reactors by combining a genome-scale metabolic reconstruction of the syngas fermentating bacterium *C. ljungdahlii* with multiphase transport equations that govern convective and dispersive processes within the spatially varying column. To obtain a computationally tractable model, we performed spatial discretization to yield a large set of ordinary differential equations (ODEs) in time with embedded linear programs (LPs). Our initial attempts to solve the discretized model within MATLAB using a straightforward combination of built-in ODE solvers and the MOSEK LP solver proved unsuccessful. We found the recently developed MATLAB based code DFBAlab [26] to be a critical enabling tool, without which this study would not have been possible. Model translation into the DFBAlab format required minimal work.

Column startup was dynamically simulated with different process parameters to generate steady-state column profiles for analysis of parameter trends. Because the liquid product stream was removed from the top of the column, we focused our analysis on liquid and gas phase concentrations at this point. Our analysis was limited to syngas feed streams containing only CO and H_2_. We predicted the following trends that could guide column design and operation for maximization of ethanol production:

- A maximum ethanol titer of 120 g/L and no acetate production were achieved at a CO mole fraction of 0.45 (Fig. 3). The ethanol concentration decreased rapidly, CO_2_ synthesis occurred and acetate quickly became the dominant byproduct at higher CO mole fractions, suggesting that H_2_ augmentation of CO-rich syngas feeds may be beneficial.
- High superficial gas velocities enhanced the ethanol titer and ethanol/acetate split at the expense of low CO and H_2_ conversions (Fig. 4), indicating the possible benefit of recycling unconsumed gas to achieve higher conversions.
- Partial recycling of unconsumed gas showed the potential to substantially improve CO and H_2_ conversions at the expense of increased gas compression costs (Fig. 6). Because recycling had the negative effect of reducing the ethanol titer and the ethanol/acetate ratio due to depleted H_2_ levels, H_2_ augmentation may be necessary to achieve acceptable process economics.
- Enhanced ethanol titer and ethanol/acetate split were achieved with increasing liquid velocity up to a critical value at which the column was washed out (results not shown). The development of reactor monitoring and control strategies would be necessary to stably operate near this critical value.
- Increasing reactor length enhanced both the ethanol titer and the ethanol/acetate split (results not shown). Because taller reactors required more syngas feed compression, an economic analysis would be needed to determine the optimal length.
- Efficient gas–liquid mass transfer was found to be critical to achieve high ethanol production and high conversions (Fig. 5). A CO mass transfer coefficient of 500 h^−1^ was predicted to produce an ethanol titer of 130 g/L, an ethanol/acetate ratio of 6, and CO and H_2_ conversions of 34 and 89 %, respectively, for a syngas feed containing 60 % CO. These results demonstrate the need for continued development of advanced bubble column designs that achieve very high gas–liquid mass transfer rates.
- The bubble column model also was used to investigate the effect of CO and H_2_ uptake parameters on reactor performance. The following trends were observed that could guide the engineering of bacterial syngas uptake kinetics for ethanol overproduction:
- Enhanced H_2_ uptake rates achieved either by increasing the maximum uptake rate or by reducing the uptake saturation constant substantially increased the ethanol titer and the ethanol/acetate ratio (Fig. 7). Consequently, *C. ljungdahlii* engineering efforts should focus on increasing H_2_ uptake rates.
- Ethanol and/or acetate inhibition of growth modeled as inhibition of the CO and H_2_ uptakes reduced biomass production but increased the ethanol titer and the ethanol/acetate ratio (results not shown). Therefore, cellular engineering efforts aimed at reducing byproduct inhibition may have limited effectiveness.
- Inhibition of CO uptake at high CO levels reduced biomass production but had almost no effect on the ethanol titer while reducing acetate synthesis (Fig. 8). Conversely, CO inhibition of H_2_ uptake reduced growth and shifted the product distribution strongly towards acetate. Consequently, *C. ljungdahlii* engineering efforts should focus on alleviating CO inhibition of H_2_ uptake.

Future work on syngas bubble column modeling could include the incorporation of more realistic column hydrodynamics [29] to improve model fidelity.

## Methods

### Model formulation

The bubble column model was formulated by combining a genome-scale metabolic reconstruction of *C. ljungdahlii* with uptake kinetics for dissolved gases and reaction-convection-dispersion type equations for gaseous and dissolved substrates and synthesized metabolic byproducts. The *C. ljungdahlii* iHN637 reconstruction accounts for 637 genes, 698 metabolites, 690 intracellular reactions, and 95 exchange reactions that capture the primary metabolic pathways involved in synthesis gas fermentation [21]. The model has been shown to produce growth on several known substrates including CO and CO_2_/H_2_ mixtures as well as to provide good agreement with experimentally determined growth and acetate production rates on fructose. Our preliminary flux balance calculations with the typical maximum growth objective showed that the primary metabolic byproducts for growth on CO/H_2_ mixtures were ethanol, acetate, and CO_2_. We assumed that the extracellular pH was maintained constant throughout the reactor such that the intracellular pH could be assumed constant at the value used for charge balancing of the metabolic reconstruction [21].

Uptake kinetics were specified for the dissolved gaseous substrates CO and H_2_ as well as for the dissolved gaseous byproduct CO_2_ that could be reassimilated. Uptake kinetics were assumed to follow inhibited Monod expressions of the form,

2 $$ {v}_i=\frac{v_{max,i}{S}_i}{K_{m,i}+{S}_i}\frac{1}{1+\frac{E+A}{K_I}} $$

where *v*_*i*_ is the uptake rate (mmol/gDW/h) of the *i*-th substrate, *S*_*i*_ is the dissolved concentration (mmol/L) of the *i*-th gaseous substrate, *v*_*max*_,_*i*_ is the maximum uptake rate, *K*_*m*_,_*i*_ is the saturation constant, and *K*_*I*_ is an inhibition constant. A combined term involving both the concentrations of ethanol (*E*) and acetate (*A*) was used to account for the known inhibitory effects of these two products on *C. ljungdahlii* growth [22, 30]. To reduce the number of model parameters, the two products were assumed to induce equal inhibition of all substrate uptake rates such that only a single *K*_*I*_ value was needed to model inhibition of growth due to high ethanol and/or acetate concentrations. Equation (2) was used to establish bounds on the possible uptake rates with the actual uptake rates being determined by the solution of the intracellular flux balance problem. Both *v*_*max*_,_*i*_ and *K*_*m*_,_*i*_ were important parameters due to the large dissolved gas concentration gradients in the bubble column reactor (see Results).

Despite synthesis gas fermentation being an active research area, we found a dearth of literature for determining the parameter values needed to calculate uptake rates for the three possible substrates (CO, H_2_, CO_2_). Parameter values for the CO maximum uptake rate and saturation constant were obtained from a recent experimental study [13]. Based on our own limited experimental data (unpublished), we assumed that the H_2_ maximum uptake rate was double the CO value. Because data was lacking for determination of the remaining parameters, the CO_2_ maximum uptake rate and the H_2_ and CO_2_ saturation constants were taken to be equal to the corresponding CO values. The value of the inhibition constant was chosen based on our previous modeling efforts involving uptake inhibition by ethanol and other toxic byproducts (Hanly and Henson 2014). Due to the large uncertainties associated with these parameter values (Table 1), we explored the sensitivity of our model predictions to the dissolved gas uptake kinetics.

**Table 1:** Nominal dissolved gas uptake parameters

Substrate	*v*_*max*_ (mmol/gDW/h)	*K*_*m*_ (mmol/L)	Source
CO	35	0.02	[13]
H_2_	70	0.02	Specified
CO_2_	35	0.02	Specified
Substrate	*K*_*I*_ (g/L)		Source
All	10		Specified

The genome-scale reconstruction of intracellular metabolism and the substrate uptake kinetics were combined with reaction-convection-dispersion type equations for the bubble column transport processes. Because our focus was describing spatially varying cellular metabolism rather than detailed modeling of the potentially complex column hydrodynamics [29], we assumed ideal plug flow for the vapor phase and plug flow plus axial dispersion for the liquid phase. These assumptions represent reasonable simplifications given the gas superficial velocities within the bubbly flow regime (<5 cm/s; [29]) and the very small liquid velocities (<0.02 cm/s) used in our simulations. Convection and dispersion were assumed to occur only in the axial direction of the bubble column reactor such that spatial variations could be captured with a single variable *z*.

The mass balance of *C. ljungdahlii* biomass had the form,

3 $$ \begin{array}{l}\frac{\partial X\left(z,t\right)}{\partial t}=\mu X-\frac{u_L}{\varepsilon_L}\frac{\partial X}{\partial z}+{D}_A\frac{\partial^2X}{\partial {z}^2}\\ {}\begin{array}{ccc}\hfill {u}_LX\left(0,t\right)-{\varepsilon}_L{D}_A\frac{\partial X\left(0,t\right)}{\partial z}=0\hfill & \hfill \frac{\partial X\left(L,t\right)}{\partial z}=0\hfill & \hfill X\left(z,0\right)={X}_0\hfill \end{array}\end{array} $$

where *X* is the biomass concentration (g/L), μ is cellular growth rates (h^−1^) obtained from the flux balance calculation, *u*_*L*_ is the liquid phase velocity, ε_L_ is the liquid phase volume fraction, and *D*_*A*_ is the axial dispersion coefficient of the liquid phase. A typical Danckwerts boundary condition was imposed at the reactor entrance (*z* = 0), while a zero slope boundary condition was applied at the reactor exit (*z* = *L*). A uniform biomass concentration profile within the reactor was used as the initial condition.

Mass balances of the dissolved gaseous substrates had the form,

4 $$ \begin{array}{l}\frac{\partial {C}_L\left(z,t\right)}{\partial t}={v}_CX+\frac{k_{m,C}}{\varepsilon_L}\left({C}^{*}-{C}_L\right)-\frac{u_L}{\varepsilon_L}\frac{\partial {C}_L}{\partial z}+{D}_A\frac{\partial^2{C}_L}{\partial {z}^2}\\ {}\begin{array}{ccc}\hfill {u}_L{C}_L\left(0,t\right)-{\varepsilon}_L{D}_A\frac{\partial {C}_L\left(0,t\right)}{\partial z}=0\hfill & \hfill \frac{\partial {C}_L\left(L,t\right)}{\partial z}=0\hfill & \hfill {C}_L\left(z,0\right)\hfill \end{array}={C}_{L0}\\ {}\frac{\partial {H}_L\left(z,t\right)}{\partial t}={v}_HX+\frac{k_{m,H}}{\varepsilon_L}\left({H}^{*}-{H}_L\right)-\frac{u_L}{\varepsilon_L}\frac{\partial {H}_L}{\partial z}+{D}_A\frac{\partial^2{H}_L}{\partial {z}^2}\\ {}\begin{array}{ccc}\hfill {u}_L{H}_L\left(0,t\right)-{\varepsilon}_L{D}_A\frac{\partial {H}_L\left(0,t\right)}{\partial z}=0\hfill & \hfill \frac{\partial {H}_L\left(L,t\right)}{\partial z}=0\hfill & \hfill {H}_L\left(z,0\right)={H}_{L0}\hfill \end{array}\end{array} $$

where *C*_*L*_ and *H*_*L*_ are the liquid phase CO and H_2_ concentrations (mmol/L), *v*_*C*_ and *v*_*H*_ are the CO and H_2_ uptake rates (mmol/gDW/h) obtained from the flux balance calculation, k_*m*, *C*_ and k_*m*, *H*_ are the corresponding gas–liquid mass transfer coefficients, and C* and H* are the saturated liquid concentrations (mmol/L) calculated from the corresponding gas phase concentrations using Henry’s law at the specified temperature and pressure. Constant gas–liquid mass transfer coefficients were used for simplicity despite their known dependence on various factors including gas bubble size [31], which was not modeled in this study. The Danckwerts boundary conditions imposed at the reactor entrance assumed the form shown since dissolved gases were not fed to the reactor, while zero slope boundary conditions were applied at the reactor exit. Uniform concentration profiles calculated from the initial gas phase concentrations using Henry’s law were imposed as initial conditions, which was consistent with the liquid phase being saturated with the feed gases prior to inoculation.

Mass balances of the two substrates in the gas phase had the form,

5 $$ \begin{array}{ccc}\hfill \frac{\partial {C}_G\left(z,t\right)}{\partial t}=-\frac{k_{m,C}}{\varepsilon_G}\left({C}^{*}-{C}_L\right)-\frac{u_G}{\varepsilon_G}\frac{\partial {C}_G}{\partial z}\hfill & \hfill {C}_G\left(0,t\right)={C}_{GF}\hfill & \hfill {C}_G\left(z,0\right)={C}_{G0}\hfill \\ {}\hfill \frac{\partial {H}_G\left(z,t\right)}{\partial t}=-\frac{k_{m,H}}{\varepsilon_G}\left({H}^{*}-{H}_L\right)-\frac{u_G}{\varepsilon_G}\frac{\partial {H}_G}{\partial z}\hfill & \hfill {H}_G\left(0,t\right)={H}_{GF}\hfill & \hfill {H}_G\left(z,0\right)={H}_{G0}\hfill \end{array} $$

where *C*_*G*_ and *H*_*G*_ are the gas phase CO and H_2_ concentrations (mmol/L), ε_G_ = 1-ε_L_ is the gas phase volume fraction, and *u*_*G*_ is the superficial gas velocity. The gas concentrations at the reactor entrance *C*_*GF*_ and *H*_*GF*_ were calculated from the partial pressures of the feed gas using the ideal gas law. Uniform initial conditions were specified by setting *C*_*G0*_ = *C*_*GF*_ and *H*_*G0*_ = *H*_*GF*_ , which again was consistent with the liquid phase being saturated with the feed gases prior to inoculation.

Mass balances on the two major metabolic byproducts ethanol and acetate had the form,

6 $$ \begin{array}{c}\hfill \frac{\partial {E}_L\left(z,t\right)}{\partial t}={M}_E{v}_EX-\frac{u_L}{\varepsilon_L}\frac{\partial {E}_L}{\partial z}+{D}_A\frac{\partial^2{E}_L}{\partial {z}^2}\hfill \\ {}\hfill \begin{array}{ccc}\hfill {u}_L{E}_L\left(0,t\right)-{\varepsilon}_L{D}_A\frac{\partial {E}_L\left(0,t\right)}{\partial z}=0\hfill & \hfill \frac{\partial {E}_L\left(L,t\right)}{\partial z}=0\hfill & \hfill {E}_L\left(z,0\right)={E}_{L0}\hfill \end{array}\hfill \\ {}\hfill \frac{\partial {A}_L\left(z,t\right)}{\partial t}={M}_A{v}_AX-\frac{u_L}{\varepsilon_L}\frac{\partial {A}_L}{\partial z}+{D}_A\frac{\partial^2{A}_L}{\partial {z}^2}\hfill \\ {}\hfill \begin{array}{ccc}\hfill {u}_L{A}_L\left(0,t\right)-{\varepsilon}_L{D}_A\frac{\partial {A}_L\left(0,t\right)}{\partial z}=0\hfill & \hfill \frac{\partial {A}_L\left(L,t\right)}{\partial z}=0\hfill & \hfill {A}_L\left(z,0\right)={A}_{L0}\hfill \end{array}\hfill \end{array} $$

where *E*_*L*_ and *A*_*L*_ are the concentrations of liquid phase ethanol (g/L) and acetate (g/L), *v*_*E*_ and *v*_*A*_ are the corresponding fluxes (mmol/L) calculated from the flux balance model, and *M*_*E*_ and *M*_*A*_ are the corresponding molecular weight (g/mmol). Gas phase balances on ethanol and acetate were omitted under the assumption of low volatility at column conditions. Danckwerts boundary conditions were imposed at the reactor entrance and zero slope boundary conditions were applied at the reactor exit as before. Uniform ethanol and acetate concentration profiles were used as initial conditions.

Mass balances on liquid and gas phase carbon dioxide had the form,

7 $$ \begin{array}{c}\hfill \frac{\partial {D}_L\left(z,t\right)}{\partial t}={v}_DX+\frac{k_{m,D}}{\varepsilon_L}\left({D}^{*}-{D}_L\right)-\frac{u_L}{\varepsilon_L}\frac{\partial {D}_L}{\partial z}+{D}_A\frac{\partial^2{D}_L}{\partial {z}^2}\hfill \\ {}\hfill \begin{array}{ccc}\hfill {u}_L{D}_L\left(0,t\right)-{\varepsilon}_L{D}_A\frac{\partial {D}_L\left(0,t\right)}{\partial z}=0\hfill & \hfill \frac{\partial {D}_L\left(L,t\right)}{\partial z}=0\hfill & \hfill {D}_L\left(z,0\right)={D}_{L0}\hfill \end{array}\hfill \\ {}\hfill \frac{\partial {D}_G\left(z,t\right)}{\partial t}=-\frac{k_{m,D}}{\varepsilon_G}\left({D}^{*}-{D}_L\right)-\frac{u_G}{\varepsilon_G}\frac{\partial {D}_L}{\partial z}\hfill \\ {}\hfill \begin{array}{cc}\hfill {D}_G\left(0,t\right)={D}_{GF}\hfill & \hfill {D}_G\left(z,0\right)={D}_{G0}\hfill \end{array}\hfill \end{array} $$

where *D*_*L*_ and *D*_*G*_ are the concentrations of liquid phase CO_2_ (mmol/L) and gas phase CO_2_ (mmol/L), *v*_*D*_ is the CO_2_ flux (mmol/L) calculated from the flux balance model, k_*m*,*D*_ is the CO_2_ gas–liquid mass transfer coefficient, and D* is the saturated liquid CO_2_ concentration (mmol/L) calculated from the corresponding gas phase concentration using Henry’s law. For liquid phase CO_2_, Danckwerts and zero slope boundary conditions were applied at the reactor entrance and exit as before. The CO_2_ concentration at the reactor entrance *D*_*GF*_ was calculated from the CO_2_ partial pressure of the feed gas using the ideal gas law. This formulation allowed CO_2_ to be a feed component and/or a metabolic byproduct. A uniform liquid phase CO_2_ concentration profile calculated from the initial CO_2_ gas phase concentration using Henry’s law was imposed as an initial condition. A uniform initial condition for gas phase CO_2_ was specified by setting *D*_*G0*_ = *D*_*GF*_.

The reactor was assumed to be isothermal, while the pressure profile was calculated from the liquid head as,

8 $$ P(z)={P}_L+{\rho}_Lg\left(L-z\right) $$

where *L* is the length of the column, ρ is the liquid phase density assumed to be equal to the density of water, and *P*_*L*_ is the pressure (Pa) at the top of the column, which was assumed to be atmospheric pressure. Accordingly, gaseous substrates were modeled to dissolve more readily in the lower portion of the column. Calculation of gas and liquid volume fractions in bubble column reactors is notoriously difficult, as the volume fractions are known to depend on a number of operating parameters [29]. The effect of the gas flow rate is known to be particularly important. Therefore, we fit gas flow rate versus gas volume fraction data [32] to a simple model [33] to derive the following relationship:

9 $$ {\varepsilon}_g=\frac{\varepsilon_{G, \max }{u}_G}{K_G+{u}_G} $$

where ε_*G*_, _*max*_ is the maximum achievable gas volume fraction and *K*_*G*_ is a type of saturation constant.

Parameter values for the bubble column reactor model were obtained from the literature to the extent possible (Tables 1 and 2). The reactor length and cross-sectional area were chosen to represent an industrial scale reactor with volume of 125,000 l and a typical length-to-diameter ratio of 10 [29]. The liquid and superficial gas velocities were chosen to achieve a liquid residence time of 100 h to maintain the gas flow in the homogeneous, bubbly regime (<5 cm/s) where dispersion effects would be small and to achieve a high gas-to-liquid velocity ratio of 300 [29]. A small value of the liquid phase dispersion coefficient was specified to improve numerical stability of the model (see “Model solution”) while ensuring that the liquid flow would be convection controlled. A feed stream with a 1.5:1 CO:H_2_ ratio and devoid of CO_2_ was used to model a CO-rich syngas mixture [9]. A very wide range of CO gas–liquid mass transfer coefficients that differ according to the reactor configuration, gas sparging method, and agitation rate have been reported [9]. We conservatively selected the CO mass transfer coefficient to be consistent with a bubble column without microsparging and internal packing. Based on the limited literature available [34], we chose the H_2_ gas–liquid mass transfer coefficient to be 250 % larger than the CO value. The CO_2_ mass transfer coefficient was specified to be equal to the CO value due to lack of data. Due to the large variability associated with these parameter values, we explored the sensitivity of our model predictions to the mass transfer coefficients. The initial conditions were chosen to mimic a newly inoculated reactor with saturated liquid compositions and no spatial gradients.

**Table 2:** Nominal parameter values for the synthesis gas bubble column reactor

Parameter	Symbol	Value	Source
Reactor length	*L*	25 m	Specified
Reactor cross-sectional area	*A*	5 m^2^	Specified
Superficial gas velocity	*u*_*G*_	75 m/h	Specified
Liquid phase velocity	*u*_*L*_	0.25 m/h	Specified
Liquid phase dispersion coefficient	*D*_*A*_	0.25 m^2^/h	Specified
Temperature	*T*	37 °C	[3]
Pressure at top of column	*P*_*L*_	1.013×10^5^ Pa	Specified
CO mole fraction in feed gas	*x*_*C*_	0.6	Specified
H_2_ mole fraction in feed gas	*x*_*H*_	0.4	Specified
CO_2_ mole fraction in feed gas	*x*_*D*_	0	Specified
CO Henry’s law constant	*H*_*C*_	8×10^−4^ mol/L/atm	[35]
H_2_ Henry’s law constant	*H*_*H*_	6.6×10^−4^ mol/L/atm	[35]
CO_2_ Henry’s law constant	*H*_*D*_	2.5×10^−2^ mol/L/atm	[35]
CO gas–liquid mass transfer coefficient	*k*_*m*_, _*C*_	80 h^−1^	[9]
H_2_ gas–liquid mass transfer coefficient	*k*_*m*_, _*H*_	200 h^−1^	[34]
CO_2_ gas–liquid mass transfer coefficient	*k*_*m*_, _*D*_	80 h^−1^	Specified
Maximum gas volume fraction	ε_*G*_, _*max*_	0.53	Fit to data
Gas volume fraction saturation constant	*K*_*G*_	540 m/h	Fit to data
Gas volume fraction	ε_*G*_	0.0646	Calculated
CO concentration at reactor entrance	*C*_*GF*_	80.64 mmol/L	Calculated
H_2_ concentration at reactor entrance	*H*_*GF*_	53.76 mmol/L	Calculated
CO_2_ concentration at reactor entrance	*D*_*GF*_	0 mmol/L	Calculated
Initial biomass concentration	*X*_*0*_	0.1 g/L	Specified
Initial gas phase CO concentration	*C*_*G0*_	80.64 mmol/L	Calculated
Initial gas phase H_2_ concentration	*H*_*G0*_	53.76 mmol/L	Calculated
Initial gas phase CO_2_ concentration	*D*_*G0*_	0 mmol/L	Calculated
Initial liquid phase CO concentration	*C*_*L0*_	1.642 mmol/L	Calculated
Initial liquid phase H_2_ concentration	*H*_*L0*_	0.903 mmol/L	Calculated
Initial liquid phase CO_2_ concentration	*D*_*L0*_	0 mmol/L	Calculated
Initial liquid phase ethanol concentration	*E*_*L0*_	0 mmol/L	Specified
Initial liquid phase acetate concentration	*A*_*L0*_	0 mmol/L	Specified

### Model solution

The bubble column reactor model is consisted of a set of PDEs for multiphase transport processes with embedded linear programs that described intracellular metabolism. We spatially discretized the PDE model using third-order finite differences for the convection terms and second-order central differences for the diffusion terms. The resulting ODE system with embedded LPs was solved with DFBAlab [26], a MATLAB code specifically designed for large-scale dynamic FBA simulations, combined with the LP solver Gurobi and the stiff ODE solver ode15s. DFBAlab requires the specification of lexicographic optimization objectives to avoid the common problem of non-unique exchange fluxes that render the ODE system impossible to integrate. The objectives were sequentially applied in the following order: (1) maximization of the growth rate, (2) maximization of the CO uptake rate, (3) maximization of the H_2_ uptake rate, (4) minimization of the CO_2_ synthesis rate, (5) minimization of the acetate synthesis rate, and (6) minimization of the ethanol synthesis rate. The ordering of these objectives had no effect on model predictions. We found that 100 spatial node points (900 ODEs, 600 LPs) provided a suitable compromise between solution accuracy and computation time. Additional details on the numerical solution procedure are available in the forthcoming publication.

## Abbreviations

DFBAlab: Dynamic flux balance analysis laboratory
FBA: Flux balance analysis
LP: Linear program
ODE: Ordinary differential equation
PDE: Partial differential equation

## References

[1] Kirkels AF, Verbong GPJ (2011). Biomass gasification: still promising? A 30-year global overview. Renew Sust Energ Rev.

[2] McKendry P (2002). Energy production from biomass (part 3): gasification technologies. Bioresour Technol.

[3] Tanner RS, Miller LM, Yang D (1993). *Clostridium ljungdahlii* sp. nov., an acetogenic species in clostridial rRNA homology group I. Int J Syst Bacteriol.

[4] Tanner RS, Wall J, Harwood C, Demain A (2008). Production of ethanol from synthesis gas. Bioenergy.

[5] Sim JH, Kamaruddin AH, Long WS, Najafpour G (2007). *Clostridium aceticum*—a potential organism in catalyzing carbon monoxide to acetic acid: application of response surface methodology. Enzyme Microb Technol.

[6] Genthner BRS, Bryant MP (1987). Additional characteristics of one-carbon-compound utilization by *Eubacterium limosum* and *Acetobacterium woodii*. Appl Environ Microbiol.

[7] Liou JSC, Balkwill DL, Drake GR, Tanner RS (2005). *Clostridium carboxidivorans* sp. nov., a solvent-producing clostridium isolated from an agricultural settling lagoon, and reclassification of the acetogen *Clostridium scatologenes* strain SL1 as *Clostridium drakei* sp. nov. Int J Syst Evol Microbiol.

[8] Daniell J, Kopke M, Simpson SD (2012). Commercial biomass syngas fermentation. Energies.

[9] Munasinghe PC, Khanal SK (2010). Biomass-derived syngas fermentation into biofuels: opportunities and challenges. Bioresour Technol.

[10] Abubackar HN, Veiga MC, Kennes C (2011). Biological conversion of carbon monoxide: rich syngas or waste gases to bioethanol. Biofuel Bioprod Bior.

[11] Riggs SS, Heindel TJ (2006). Measuring carbon monoxide gas–liquid mass transfer in a stirred tank reactor for syngas fermentation. Biotechnol Prog.

[12] Hurst KM, Lewis RS (2010). Carbon monoxide partial pressure effects on the metabolic process of syngas fermentation. Biochem Eng J.

[13] Mohammadi M, Mohamed AR, Najafpour GD, Younesi H, Uzir MH (2014). Kinetic studies on fermentative production of biofuel from synthesis gas using *Clostridium ljungdahlii*. Sci World J.

[14] Datar RP, Shenkman RM, Cateni BG, Huhnke RL, Lewis RS (2004). Fermentation of biomass-generated producer gas to ethanol. Biotechnol Bioeng.

[15] Henstra AM, Sipma J, Rinzema A, Stams AJM (2007). Microbiology of synthesis gas fermentation for biofuel production. Curr Opin Biotechnol.

[16] Papin JA, Price ND, Wiback SJ, Fell DA, Palsson BO (2003). Metabolic pathways in the post-genome era. Trends Biochem Sci.

[17] Hanly TJ, Henson MA (2011). Dynamic flux balance modeling of microbial co-cultures for efficient batch fermentation of glucose and xylose mixtures. Biotechnol Bioeng.

[18] Hjersted JL, Henson MA, Mahadevan R (2007). Genome-scale analysis of *Saccharomyces cerevisiae* metabolism and ethanol production in fed-batch culture. Biotechnol Bioeng.

[19] Varma A, Palsson BO (1994). Stoichiometric flux balance models quantitatively predict growth and metabolic by-product secretion in wild-type *Escherichia coli* W3110. Appl Environ Microbiol.

[20] Mahadevan R, Edwards JS, Doyle FJ (2002). Dynamic flux balance analysis of diauxic growth in *Escherichia coli*. Biophys J.

[21] Nagarajan H, Sahin M, Nogales J, Latif H, Lovley DR, Ebrahim A (2013). Characterizing acetogenic metabolism using a genome-scale metabolic reconstruction of *Clostridium ljungdahlii*. Microb Cell Fact..

[22] Younesi H, Najafpour G, Mohamed AR (2005). Ethanol and acetate production from synthesis gas via fermentation processes using anaerobic bacterium, *Clostridium ljungdahlii*. Biochem Eng J..

[23] Harcombe WR, Riehl WJ, Dukovski I, Granger BR, Betts A, Lang AH (2014). Metabolic resource allocation in individual microbes determines ecosystem interactions and spatial dynamics. Cell Rep.

[24] Fang Y, Scheibe TD, Mahadevan R, Garg S, Long PE, Lovley DR (2011). Direct coupling of a genome-scale microbial in silico model and a groundwater reactive transport model. J Contam Hydrol.

[25] Jayasinghe N, Franks A, Nevin KP, Mahadevan R (2014). Metabolic modeling of spatial heterogeneity of biofilms in microbial fuel cells reveals substrate limitations in electrical current generation. Biotechnol J.

[26] Gomez JA, Höffner K, Barton PI (2014). DFBAlab: a fast and reliable MATLAB code for dynamic flux balance analysis. BMC Bioinformatics..

[27] Gaddy JL, Arora DK, Ko CW, Philip JR, Basu R. Methods for increasing the production of ethanol from microbial fermentation. US Patent Application 7285402 B2.

[28] Munasinghe PC, Khanal SK (2010). Syngas fermentation to biofuel: evaluation of carbon monoxide mass transfer coefficient (k (L) a) in different reactor configurations. Biotechnol Prog.

[29] Kantarci N, Borak F, Ulgen KO (2005). Bubble column reactors. Process Biochem.

[30] Drake HL, Gossner AS, Daniel SL (2008). Old acetogens, new light. Ann Ny Acad Sci..

[31] Bouaifi M, Hebrard G, Bastoul D, Roustan M (2001). A comparative study of gas hold-up, bubble size, interfacial area and mass transfer coefficients in stirred gas–liquid reactors and bubble columns. Chem Eng Process.

[32] Prakash A, Margaritis A, Li H, Bergougnou MA (2001). Hydrodynamics and local heat transfer measurements in a bubble column with suspension of yeast. Biochem Eng J.

[33] Joshi JB, Sharma MM (1979). Circulation cell model for bubble-columns. T I Chem Eng-Lond.

[34] Bredwell MD, Srivastava P, Worden RM (1999). Reactor design issues for synthesis-gas fermentations. Biotechnol Prog.

[35] Linstrom PJ, Mallard WG (2015). NIST Chemistry WebBook, NIST standard reference database number 69.

